# Model-driven diabetes care: study protocol for a randomized controlled trial

**DOI:** 10.1186/1745-6215-14-139

**Published:** 2013-05-14

**Authors:** Stein Olav Skrøvseth, Eirik Årsand, Fred Godtliebsen, Ragnar M Joakimsen

**Affiliations:** 1 Norwegian Centre for Integrated Care and Telemedicine, University Hospital of North Norway, Tromsø, 9038, Norway; 2 Department of Computer Science, University of Tromsø, Tromsø, 9037, Norway; 3 Department of Mathematics and Statistics, University of Tromsø, Tromsø, 9037, Norway; 4 Division of Internal Medicine, University Hospital of North Norway, Tromsø, 9038, Norway; 5 Department of Clinical Medicine, University of Tromsø, Tromsø, 9037, Norway

**Keywords:** Type 1 diabetes, Mobile phones, Data-driven feedback, Self-measured blood glucose, Pattern recognition

## Abstract

**Background:**

People with type 1 diabetes who use electronic self-help tools register a large amount of information about their disease on their participating devices; however, this information is rarely utilized beyond the immediate investigation. We have developed a diabetes diary for mobile phones and a statistics-based feedback module, which we have named Diastat, to give data-driven feedback to the patient based on their own data.

**Method:**

In this study, up to 40 participants will be given a smartphone on which is loaded a diabetes self-help application (app), the Few Touch Application (FTA). Participants will be randomized into two groups to be given access to Diastat 4 or 12 weeks, respectively after receiving the smartphone, and will use the FTA with Diastat for 8 weeks after this point. The primary endpoint is the frequency of high and low blood-glucose measurements.

**Discussion:**

The study will investigate the effect of data-driven feedback to patients. Our hypothesis is that this will improve glycemic control and reduce variability. The endpoints are robust indicators that can be assembled with minimal effort by the patient beyond normal routine.

**Trial registration:**

Clinicaltrials.gov: NCT01774149

## Background

Patients with type 1 diabetes (T1D) on self-measured blood glucose (SMBG) regimens need to manage a complex and challenging condition, particularly when deciding their insulin administration. The interactions between many variables means that the decision-making process is difficult and leads to poorly regulated blood glucose for many patients [1]. Patients who use electronic aids such as blood-glucose monitors and mobile phone applications (apps) gather large amounts of data on their own disease on these units. The goal of our study is to investigate whether statistical analysis and visualization of data on mobile phones leads to better control of blood glucose for adults with T1D. We have developed a mobile phone-based application (app) for the Android platform called the Few Touch Application (FTA), and an additional module for data analysis, called Diastat. .The hypothesis is that giving this type of data-driven feedback to patients will improve glycemic control.

Diastat was developed based on knowledge from a previous data-gathering study performed in 2011, in which 30 patients used an earlier version of the FTA for 3 months. Of these 30 patients, 18 used the application actively, and thus allowed us to gather data on which to build the algorithms to be tested in this study [2].

Diastat relies in part on precise carbohydrate counting and recording. This is not commonly applied rigorously by diabetes patients in Norway, and thus there is a need to educate patients about monitoring their carbohydrate intake. There is evidence that carbohydrate counting itself improves glycemic control [3,4], and this study does not aim to test this. Thus, to remove this as a potential confounder, all enrolled patients will receive training in carbohydrate counting at the beginning of the study, and the study will use a stepped-wedge design with two groups, to ensure that the motivation for counting is equal in both groups.

Long-term blood-glucose level as measured by glycated hemoglobin (HbA1c) is commonly used as an indicator of how well regulated a patient’s blood-glucose levels is. Recently, attention has been focused on glucose variability as a complementary indicator of glucose control [5]. Variability is difficult to define precisely in SMBG, but the rate of low and high measurements beyond given thresholds is a reasonably good, simple, and robust indicator of glycemic control, as it is directly related to other measures of glycemic variability such as low or high blood glucose index [6].

## Design

**Figure 1:**
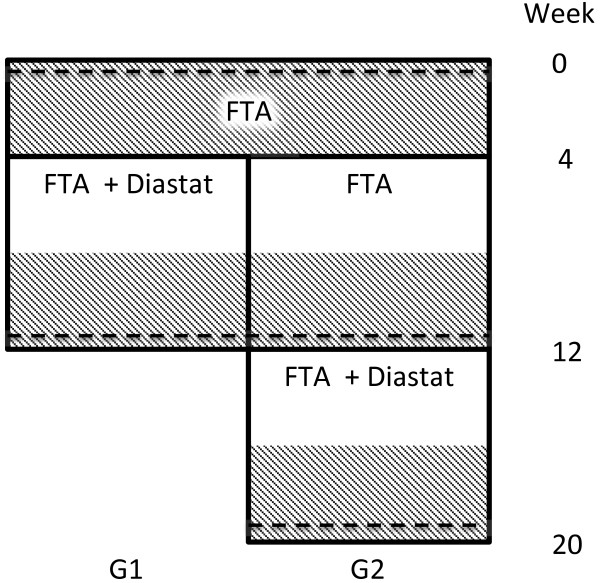
**Patient flow in the study.** HbA1c measurements indicated by dashed lines, endpoint monitoring periods are shaded.

The study is a two-group, stepped-wedge randomized controlled trial [7], with no blinding of participants. Participants will be randomized into two equal size groups, G1 and G2. Both groups will run the FTA without Diastat for 4 weeks, after which Diastat will be turned on for G1. After 8 more weeks, G2 will also receive access to Diastat. Both groups will run the FTA with Diastat for 8 weeks, so that G1 will finish the study in week 12 and G2 will finish in week 20. Data registered on the phone consists of blood-glucose measurements, insulin administrations, carbohydrate intake, physical activity, and comments, and all data registered on the phone will be automatically transferred to a secure server. The study design and patient flow is summarized in Figure 1. The primary comparison points are at the start of study and after 12 weeks when G1 will have used Diastat for 8 weeks, and the other group is just starting its use. Thus, adjusting for repeated time points is not a concern in the sample-size calculation [8].

The study was submitted for approval by the regional ethics committee (REC North, case no. 2011/1939), who waived the study as not required for submission. The Norwegian data inspectorate approved the data handling protocol on 28 January 2013.

### Randomization

After the first meeting, the study population will be randomized by assigning a number to each participant, and then randomizing using the sample command in the R software [9]. Randomization will not be stratified.

### Study population

Participants will be recruited from all eligible patients registered at the Division of Internal Medicine, University Hospital of North Norway (UNN). Inclusion criteria are that patients must: be older than 18 years; have had a diagnosis of T1D for at least 1 year; and have basic familiarity with mobile phones, and use a mobile phone on a daily basis.

Exclusion criteria are: pregnancy; inability to understand or conform to the guidelines when presented with the app; and severe complications attributed to the diabetes that would render participation unethical or medically challenging, as determined by the participating physician. Use of insulin pumps or continuous glucose meters is not an exclusion criterion in itself, but the patient must commit to use FTA and traditional SMBG as part of their treatment in the study period.

### Intervention

All participants will be offered a mobile phone with FTA installed. A participant can choose to use their own phone on condition that it is compatible, that is, that it runs Android version 2.3 or later, has a screen resolution at least 480 × 800 pixels and is Bluetooth-enabled. Phones not previously tested with the application must be certified as suitable. Participants choosing to use their own phone will receive compensation if they complete the study. All participants who complete the study can keep the phones and app, and continue usage after the study ends, but technical support will be limited after this point.

Participants will also be given a blood glucose meter (OneTouch® UltraMini®; LifeScan Inc., Milpitas, CA, USA) and a connected Bluetooth adapter (Polytel® Glucose Meter Accessory; Polymap Wireless, Tuscon, AZ, USA) that is configured for their phone. Configuration will happen either before the initial meeting for the provided handsets or will occur on site at the initial meeting if the participant chooses to use their own phone.

All patients will be instructed to measure their blood glucose with the provided blood glucose monitoring system at least five times per day, and when suspecting hypoglycemia or hyperglycemia. Because data are automatically transferred to the secure server, the researchers will be able to monitor the data for safety issues. Although this is considered unlikely, we can thus ensure that the intervention does not significantly increase the chance of severe hypoglycemic events.

### Initial meeting

At the initial meeting, all patients will be informed in detail about the study, will receive training in carbohydrate counting by a nutrition specialist, and will be shown by the project team how to use the app. Participants will then be given a phone with FTA pre-installed, or for those participants who prefer to use their own phones, FTA will be installed for them. Participation at this initial seminar is required for study inclusion, and patients will be required to sign a consent form of consent and forms outlining the conditions under which they receive the provided equipment.

### Follow-up meeting

All participants will attend a follow-up meeting before Diastat is activated on their phone. During this meeting, participants will be able to give feedback on the application, and they will be retrained in use of the Diastat module. HbA1c will also be measured at this meeting for the G2 group.

### Diastat

The Diastat module consists of three separate parts, which are briefly described below.

#### Periodicity

Periodicity is the ‘typical’ blood glucose over 24 hours and over 1 week, and is shown graphically along with uncertainty estimates. The calculated 24-hour value and variation is shown on the app's main page.

#### Trend

Multi-scale analysis of registered blood glucose is performed using the cSiZer-method [10], and if there is a significant trend on any scale, this is shown in the graphic trend view. Any current, short-scale (less than 48 hours) trend is shown on the main page.

#### Situation matching

When participants record their insulin injection, they can see a list of similar situations to the current situation, along with subsequent blood-glucose readings in those situations [11]. Those situations are matched and ranked based on available data in the application and a dynamic metric on those data. Thus patients have a case-based reasoning tool to aid in the decision making when administering insulin.

### Recruitment

All patients will be recruited at the Division of Internal Medicine at UNN, where all eligible patients are shown a presentation of the project on a tablet and given an information letter during scheduled visits. If they agree to participation, their participating information will be delivered to the principal investigator at planned intervals, and they will be invited to the 2-day initial seminar.

### Outcome measures

Primary outcome is number of low and high blood-glucose measurements per week. The limits for high and low blood glucose have been determined based on the results from the previous study, so that there should be on average a similar frequency of high and low readings. Based on the data from the previous study [2] and the frequency of the respective events, we have defined the normal range as 4–15 mmol/l, which provides for an equal number of events (roughly 5–6 events per week) on each side of this range. Patients will be encouraged to measure their blood glucose whenever they suspect high or low values, and to measure at least five times per day.

Blood-glucose data will be transferred automatically via a Bluetooth adapter from the glucose meter to the phone, and from there to a secure server to which the researchers have access. Thus, there will be no need for any special action by the participants to obtain the outcome.

Secondary outcomes are: 1) HbA1c measured at the initial seminar, the follow-up meeting, and the end of study; and 2) participant satisfaction with the application, measured by a questionnaire at the end of study, using the System Usability Scale [12]. Patient empowerment will also be measured using the Diabetes Empowerment Scale-Short Form [13] at the start and end of the study.

### Sample-size calculation

Sample size is calculated based on number of hypoglycemic events, with a significance level of 5% and power of 80%. We assume that for any patient, the rate of events during a time period follows a Poisson distribution, which is supported by the previous study data. A 20% reduction in the rate of hypoglycemic events per time unit requires a time-sample size of *Nt* = 353/*r*, where *r* is the baseline rate [14]. Assuming a baseline rate of *r* = 6 events/week, we arrive at 59 participant-weeks in each group, or for a 4-week surveillance period, 15 participants per group. To account for withdrawal and some participants having low utilization of the equipment, we are aiming for a total of 40 participants.

### Analysis

The endpoints will be analyzed per protocol with standard statistical tests using the standard R software package [9]. Additionally, intention-to-treat analysis will be performed. Participants not compliant with the study protocol (for example, not registering blood glucose to a sufficient degree) will be excluded from the analysis. Specifically, anyone with fewer than 4 glucose measurements for less than 50% of their participating days will be excluded.

## Discussion

The study will, using delayed start, be able to detect changes in the primary endpoint and HbA1c both between the groups in the first 3 months, and by using the patients in G2 as their own control. Controlling blood glucose to a sufficient degree is a major challenge for patients with T1D, and the study will investigate if giving advanced data-driven feedback of this type can provide better control. Variability in blood glucose is considered an important factor for glucose management, complementary to HbA1c. Thus, the primary endpoint in this study aims to measure precisely the variability rather than the long-term blood glucose. Using HbA1c and questionnaires allows us to obtain more complete information on the effect on the patients.

Assessing variability using SMBG measurements is difficult, and the measures we have chosen could be improved upon. However, the measures are robust indicators, which, together with the automatic transfer of values, should ensure that we get a complete picture with minimal intervention by the participants. The choice of number of events as outcome mean thats a realistic sample size can be acquired. The number of events would realistically depend on the number of measurements, but by requiring a minimum threshold of events, and assuming a non-substantial variation in this, the numbers will nevertheless represent a clinically relevant outcome.

An essential component of the situation matching is the carbohydrate counting. Patients in Norway are typically not accustomed to carbohydrate counting, and need to be educated in how to do this. Hence, all participants will receive training in carbohydrate counting at the start of the study. However, whether the training will be adequate for the study is uncertain, and the results of the study must be interpreted in this light when applied to regions or countries where carbohydrate counting is more widespread.

The randomization will not be stratified according to use of pump or continuous glucose meter (CGM). This is a potential confounder in the study, but the analysis can be stratified by pump/CGM use. In addition, the relatively low sample size of the study means that stratification might lead to under-populated subgroups.

The risk of adverse events in this study is considered to be negligible, and therefore safety monitoring is not planned. However, the server data will be accessible to the team throughout the study, thus if safety concerns are raised, the data can be investigated.

Diastat requires a significant learning curve, which may inhibit efficient usage. However, we believe that there is a need for patient education and understanding of their condition, and that people with complex and serious conditions such as T1D are motivated to learn techniques that can improve their disease management. The suggested and studied approach focusing on greater participant involvement in disease management is ambitious, but in line with a recent large increase in patient-oriented apps and services. We believe this change is necessary to meet the coming scarcity of healthcare resources, and to make a substantial positive change in the health outcome of patients with diabetes.

## Trial status

At the time of submission, the trial was at the planning stage and had not yet started recruiting patients.

## Abbreviations

CGM: Continuous glucose monitor; FTA: Few Touch Application; HbA1c: Hemoglobin A1c; MDDC: Model-driven diabetes care; SMBG: Self-measured blood glucose; T1D: Type 1 diabetes; UNN: University Hospital of North Norway.

## Competing interests

The authors declare that they have no competing interests.

## Authors’ contributions

SOS and EÅ conceived the study. All authors contributed to the design and planning of the study. All authors read and approved the final manuscript.
